# Learning the Cost Function for Foothold Selection in a Quadruped Robot †

**DOI:** 10.3390/s19061292

**Published:** 2019-03-14

**Authors:** Xingdong Li, Hewei Gao, Fusheng Zha, Jian Li, Yangwei Wang, Yanling Guo, Xin Wang

**Affiliations:** 1 College of Mechanical and Electrical Engineering, Northeast Forestry University, Harbin 150040, China; lixd@nefu.edu.cn (X.L.); 15146637523@nefu.edu.cn (H.G.); wang.yangwei@nefu.edu.cn (Y.W.); nefugyl@nefu.edu.cn (Y.G.); 2 State Key Laboratory of Robotics and System (HIT), Harbin 150080, China; 3 Shenzhen Academy of Aerospace Technology, Shenzhen 518057, China; xin.wang@chinasaat.com

**Keywords:** quadruped robot, foothold selection, TOF camera, 2.5D elevation map, supporting vector machine

## Abstract

This paper is focused on designing a cost function of selecting a foothold for a physical quadruped robot walking on rough terrain. The quadruped robot is modeled with Denavit–Hartenberg (DH) parameters, and then a default foothold is defined based on the model. Time of Flight (TOF) camera is used to perceive terrain information and construct a 2.5D elevation map, on which the terrain features are detected. The cost function is defined as the weighted sum of several elements including terrain features and some features on the relative pose between the default foothold and other candidates. It is nearly impossible to hand-code the weight vector of the function, so the weights are learned using Supporting Vector Machine (SVM) techniques, and the training data set is generated from the 2.5D elevation map of a real terrain under the guidance of experts. Four candidate footholds around the default foothold are randomly sampled, and the expert gives the order of such four candidates by rotating and scaling the view for seeing clearly. Lastly, the learned cost function is used to select a suitable foothold and drive the quadruped robot to walk autonomously across the rough terrain with wooden steps. Comparing to the approach with the original standard static gait, the proposed cost function shows better performance.

## 1. Introduction

To operate autonomously on a rough terrain, a quadruped robot has to perceive the environment, build the terrain model, localize itself and plan its path and gait. There are several types of terrain models that can be used for a mobile robot, such as raw point cloud, octree [1], multi-level surface (MLS) maps [2] and a 2.5D elevation map [3], etc. The 2.5D elevation map is chosen as the model for selecting footholds in this paper, and several frames from TOF camera are captured and registered [4,5,6] to generate the complete point cloud of the terrain, and then the elevation map is constructed based on the cloud. In order to improve the map, measuring error [7] of the TOF camera should be reduced as much as possible, and some new technologies [8] can be applied to increase efficiency of the map generation. Selecting a suitable foothold on the 2.5D elevation map for each foot is a key technology, which will take terrain features, robot trunk pose and collision between leg and terrain, etc. into consideration. It is nearly impossible to adjust the parameters of the foothold selection system, and using machine learning techniques seems unavoidable.

There have been so many impressive works [9,10,11,12,13,14] to autonomously plan a legged robot and select a proper foothold on rough terrains. Barasuol et al. [9] proposed an algorithm for reactive trotting with foot placement corrections through visual pattern classification, it only classifies the terrain, but it did not evaluate the terrain complexity for each candidate foothold because it is designed for quadruped trotting with the help of Motion Capture System. Fankhauser et al. [10] designed a foothold score function that is a linear of different quality measures including only terrain features, for example, the slope, curvature and roughness, and so on. Mastalli et al. [11] decomposed the quadruped robot walking problem into body action planning and footstep planning, in the part of footstep planning, a cost function is also defined that considered terrain features and robot body stability, although the default footholds were not employed in their approach. Central Pattern Generators (CPGs) [12] are often used to design a controlling system [13,14] for quadruped robots, which simulate the animal walking regularity. In general application of CPGs [14] on quadruped robots, CPGs is responsible for generating torque series to adjust the robot pose and leg. Another branch of the research on autonomously controlling quadruped robot on rough terrain is based on learning related techniques. A large number of excellent works [15,16,17] are spawned as a result of DARPA’s project [18] concentrating on developing an intelligent algorithm on learning locomotion with a LittleDog [19] platform. Many of these works assumed a known model of the environment, and nearly perfect pose estimation using a motion capture system. Only few works [20] plan the Littledog to walk on rough terrain in real time.

Let us be focused on the problem of selecting a foothold for the quadruped robot. In this paper, a cost function of selecting foothold is designed and learned for a real quadruped robot driven by hydraulic pressure. In fact, the idea of selecting footholds for quadruped robots has been introduced in our previous conference paper [21], which was published in the proceedings of the 2nd International Conference on Advanced Robotics and Mechatronics, but that paper mainly described the approach for computing terrain features in simulation environments. This paper is focused on applying the foothold selection idea on the real quadruped robot, including modelling the real robot and learning parameters for foothold selection on the real terrain.The parameters for selecting foothold are optimized using an expert based learning technique, and the training data set are acquired by constructing the 2.5D elevation map of real physical terrain using a TOF camera. There are some similar works that learn to select foothold for legged robot, such as Zico Kolter et al. [16], select the best foothold from several randomly sampled candidate footholds on the point cloud map to generate a training set for learning cost function, given that the terrain information is fully known. Belter et al. [22] learned a decision surface that can classify the terrain to distinguish between the good and the poor footholds, although the training set is generated only from a simulation environment. In fact, the idea of this paper comes from an excellent work [23], which also introduced the default foothold for selecting a suitable foothold. This idea was carried out in a simulation environment with a structured terrain, and only the candidate footholds on the front or back of the default foothold are selected, so some better candidate footholds on the right or left of the default foothold may be missed out.

The remainder of the paper is organized as follows: Section 2 presents the model of the physical quadruped robot, and introduces default foothold based on the model; Section 3 describes the algorithms to compute terrain features contributing the cost function; Section 4 defines the cost function of selecting a foothold, whose inputs include several types of features; Section 5 describes how to generate training data set, and the parameters of the cost function are learned using such a data set; Section 6 will apply the learned cost function to the real quadruped robot to navigate it across a rough terrain composed of several wooden steps; the last Section 7 will conclude the whole paper.

## 2. Modelling the Quadruped Robot

### 2.1. DH Parameters of the Model

**Figure 1:**
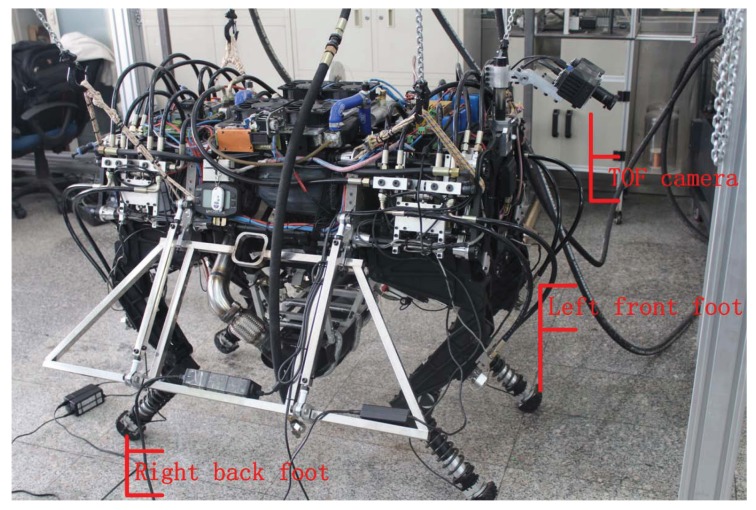
Physical platform of the quadruped robot.

The cost of selecting a foothold is a function whose inputs include relative pose between default foothold and that candidate foothold, in order to compute default foothold, the model of the quadruped robot shown in Figure 1 is represented with DH parameters. There are three degrees for each leg of the robot, which are driven by three hydraulic cylinders, respectively.

**Figure 2:**
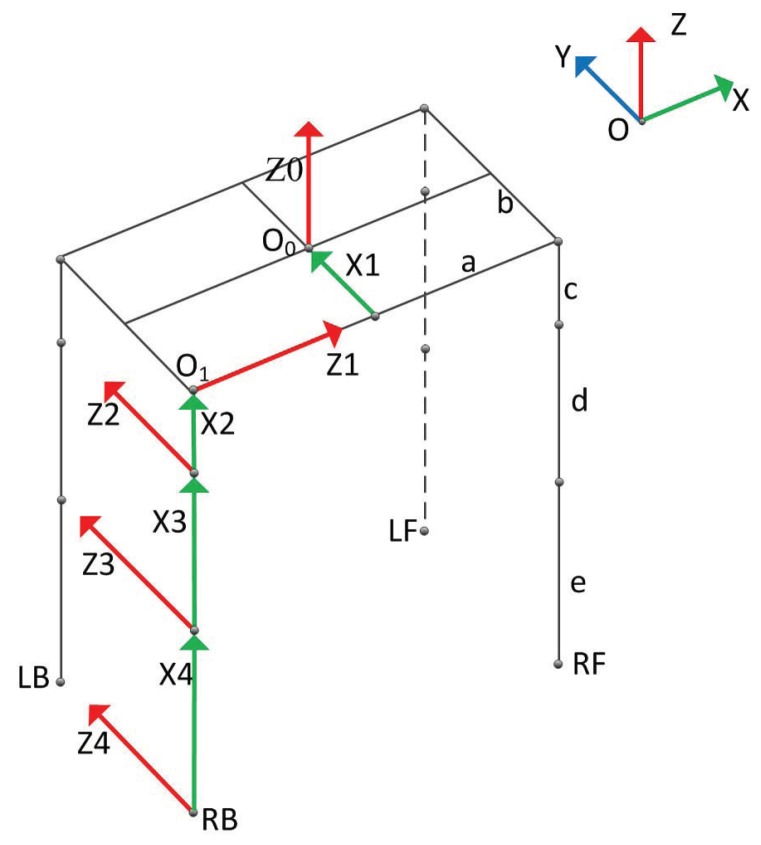
Kinematic model of the quadruped robot.

A kinematic model of the robot is shown in Figure 2, and $Z_{1},Z_{1},Z_{3}$ are the rotational axes for three joint angles of the right back leg. *a* is the half length of robot body, *b* is the half width of robot body, *c* indicates the length of the connecting rod between the body and thigh, *d* is the length of thigh and *e* is the length of shank, these constant parameters are listed in Table 1. Taking the right back leg as an example, the red arrow line represents a rotation axis (Z), and the green arrow line represents a common vertical line between two rotational axises, whose direction is equal to that of the axis X. $Z_{0}$ and $Z_{4}$ are two virtual axes introduced for convenient representation, but there is no rotational degree of freedom actually. $Z_{0}$ lies at the center of robot body, and it is supposed that $X_{0}$ and $X_{1}$ share the same line.

Taking the right back leg as the example, let $\alpha^{RB},\beta^{RB},\gamma^{RB}$ represent degrees w.r.t. side-sway joint (around $Z_{1}$ axis), hip joint and knee joint, respectively. The sign of the angle is determined in terms of the right-hand screw rule, and their ranges are listed in Table 2.

DH parameters of the model will be computed for resolving inverse kinematic of the foot, all the parameters are shown in Table 3. Kinematics equation of right the back foot is represented as Equation (1).

(1) $$\left\{\begin{array}{c} X_{RB}=d\cos\left(\beta^{RB}\right)-e\cos\left(\gamma^{RB}-\beta^{RB}\right),\\ Y_{RB}=\left(d\sin\left(\beta^{RB}\right)+e\sin\left(\gamma^{RB}-\beta^{RB}\right)+c\right)\sin\left(\alpha^{RB}\right),\\ Z_{RB}=-\left(d\sin\left(\beta^{RB}\right)+e\sin\left(\gamma^{RB}-\beta^{RB}\right)+c\right)\cos\left(\alpha^{RB}\right). \end{array}\right.$$

### 2.2. Default Foothold

**Figure 3:**
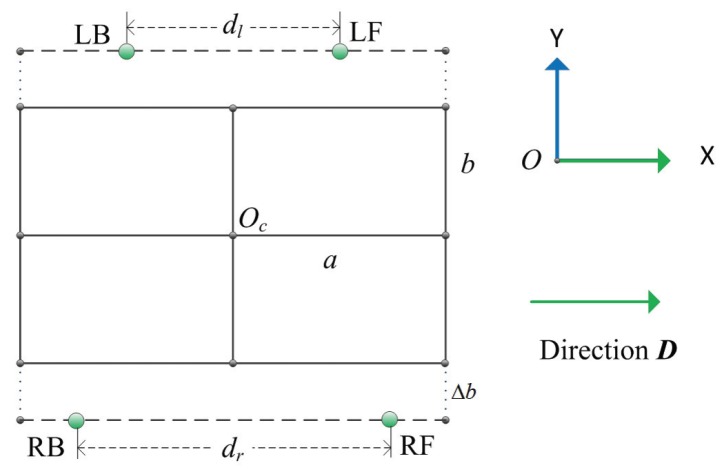
Default foothold of the quadruped robot.

The default foothold in the 2D plane is computed based on the body center of the robot and the movement direction, which is the default position of the foot before walking on flat terrain with a standard static gait. The model in Figure 2 is projected onto a 2D plane, which is shown in Figure 3, and the green point represents the default position of feet. LF, RF, LB and RB stand for the default positions of left front foot, right front foot, left back foot and right back foot, respectively. Two default footholds on the same side are exactly symmetric, the distance between two left footholds is $d_{l}$, and the distance of that on the right is $d_{l}$. Two distances must satisfy the following inequality constraint in Equation (2); in the following experiment, $d_{l}$ and $d_{r}$ is set to 0.96 m and 1.02 m, respectively.

(2) $$0<d_{l}\leq d_{r}\leq 2a.$$

In order to increase the stability of the robot, the default footholds are selected outside the robot body coverage area by controlling the rotation angle around the axis $Z_{1}$ in Figure 2, which can increase the supporting triangle area. The distance between the default footholds from two sides is larger than the width of the robot body, which is measured by 2Δb whose range is represented by Equation (3), where *h* is the default height of the robot body from the horizontal ground, and $42.6426^{\circ }$ is the maximized angle around the $Z_{1}$ axis. Δb is set to 0.125 m in the experiment:

(3) $$0<\Delta b\leq h\times tan(42.6426^{\circ }).$$

Default footholds are computed using the Equation (4), where $R_{2d}\left(\alpha \right)$ is the rotational matrix around the vertical axis in 2D space shown in Equation (5):

(4) $$\left\{\begin{array}{c} F_{lf}=O_{c}+\sqrt{{d_{l}}^{2}/4+{\left(b+\Delta b\right)}^{2}}R_{2d}\left(\alpha_{lf}\right)D,\\ F_{rf}=O_{c}+\sqrt{{d_{r}}^{2}/4+{\left(b+\Delta b\right)}^{2}}R_{2d}\left(\alpha_{rf}\right)D,\\ F_{lb}=O_{c}+\sqrt{{d_{l}}^{2}/4+{\left(b+\Delta b\right)}^{2}}R_{2d}\left(\alpha_{lb}\right)D,\\ F_{rb}=O_{c}+\sqrt{{d_{r}}^{2}/4+{\left(b+\Delta b\right)}^{2}}R_{2d}\left(\alpha_{rb}\right)D, \end{array}\right.$$

(5) $$R_{2d}\left(\alpha \right)=\left[\begin{array}{cc} cos(\alpha ) & \;\;\;-sin(\alpha )\\ sin(\alpha ) & \;\;\;cos(\alpha ) \end{array}\right].$$

## 3. Computing the Terrain Features

Suppose there has been a complete point cloud map that can be constructed with the Simultaneous Localization and Mapping (SLAM) algorithms [4,5,6]. In our robot, two data frames are transformed to the same coordinate system and then they are combined [4], and poses of over two frames must be optimized for decreasing the combining error [5,6]; a 2.5D elevation map is firstly constructed from the point cloud map, and then terrain features around the foothold are extracted [21].

### 3.1. Constructing 2.5D Elevation Map Based on Grids

**Figure 4:**
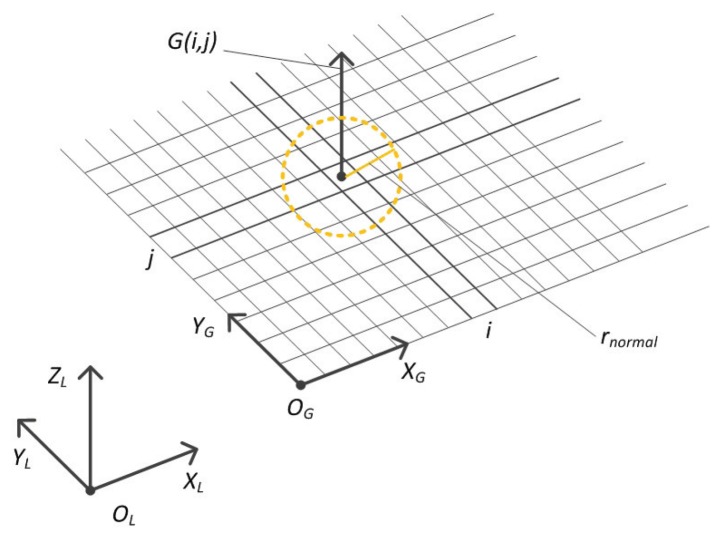
Structure of the 2.5D elevation map.

After 3D point clouds are generated from several TOF frames using such SLAM algorithms [4,5,6], all of the 3D points in the cloud are transformed into the specified referencing coordinate system whose Z-axis is antiparallel to the gravity direction. The coordinate system is called $O_{L}-X_{L}Y_{L}Z_{L}$ shown in Figure 4, and then the elevation map is constructed in this coordinate system, and the smallest unit for selecting foothold is one grid.

G(i,j) is the elevation at the grid (i,j), $r_{normal}$ is the radius for searching 3D points around the grid center (the points inside the dotted yellow circle), normal and curvature of the terrain are computed from such resulting 3D points, which are used to evaluate the cost of selecting the foothold centered at the grid G(i,j). $r_{normal}$ should be adjusted properly according to the foot size.

Let $P_{L}$ be the set of the points located in the coordinate system $O_{L}-X_{L}Y_{L}Z_{L}$:

(6) $$P_{L}=\left\{p_{1}\left(x_{1},y_{1},z_{1}\right),p_{2}\left(x_{2},y_{2},z_{2}\right),\ldots ,p_{n}\left(x_{n},y_{n},z_{n}\right)\right\}.$$

The points in $P_{L}$ are projected and partitioned into the 2D grid according to the (x,y) coordinate of the point, the start point of the grid is the origin of 2D coordinate system $O_{G}-X_{G}Y_{G}$. $O_{L}-X_{L}Y_{L}$ is transformed to $O_{G}-X_{G}Y_{G}$ by the translation vector $t_{L-G}\left(t_{x},t_{y},\delta_{z}\right)$. The elevation of the grid is computed by referencing the plane $O_{G}-X_{G}Y_{G}$. $\delta_{z}$ represents the translation in the vertical direction. $t_{x},t_{y}$ in the vector $t_{L-G}$ are the minimum values of *x* and *y* in the point set $P_{L}$:

(7) $$\left\{\begin{array}{c} t_{x}=x_{\min}=\min\left\{x_{1},x_{2},\ldots ,x_{n}\right\},\\ t_{y}=y_{\min}=\min\left\{y_{1},y_{2},\ldots ,y_{n}\right\}. \end{array}\right.$$

The resolution of the grid map is $\left(\Delta_{x},\Delta_{y}\right)$; suppose there are *m* points located inside the grid (i,j), constituting the subset $P_{L}^{ij}\subset P_{L}$:

(8) $$P_{L}^{ij}=\left\{{p_{k}}_{{}_{1}},{p_{k}}_{{}_{2}},\ldots ,p_{k_{m}}\right\}.$$

For any point in the subset $P_{L}^{ij}$, $\forall {p_{k}}_{{}_{r}}\in P_{L}^{ij}$, the coordinate w.r.t both *x* and *y* dimensions must satisfy the following constraints:

(9) $$\left\{\begin{array}{c} x_{\min}+\left(i-1\right)\times \Delta_{x}\leq {x_{k}}_{{}_{r}}<x_{\min}+i\times \Delta_{x},\\ y_{\min}+\left(j-1\right)\times \Delta_{y}\leq {y_{k}}_{{}_{r}}<y_{\min}+j\times \Delta_{y}. \end{array}\right.$$

Elevation of the grid (i,j) is computed by averaging *Z* coordinates of all the points in $P_{L}^{ij}$; supposing that there are *m* points falling into such a grid, then the elevation can be computed using the following equation:

(10) $$G\left(i,j\right)=\frac{\sum_{r=1}^{m}z_{k_{r}}}{m}.$$

The flag bit is introduced to indicate whether there is valid point in the grid or not, and the bit is represented by $V_{G}$:

(11) $$V_{G}\left(i,j\right)=\left\{\begin{array}{c} 1,\;\;\mathrm{If}\;|P_{L}^{ij}|>0,\\ 0,\;\;\mathrm{If}\;|P_{L}^{ij}|=0. \end{array}\right.$$

### 3.2. Computing Normal and Curvature

**Figure 5:**
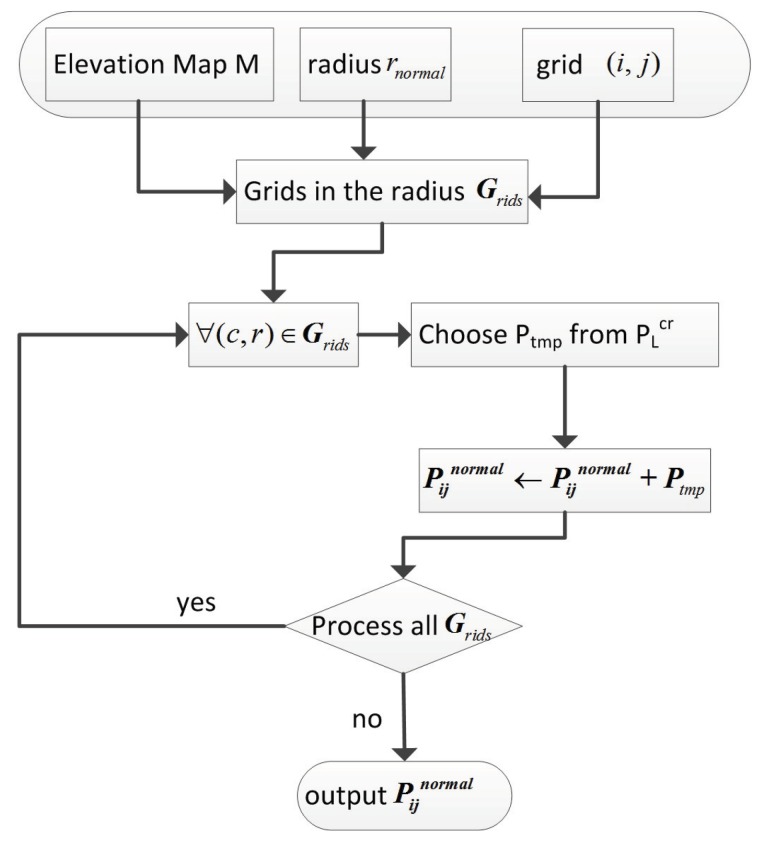
Flow chart of the algorithm for searching points around some grid.

Normal and curvature are computed directly from 3D points. In order to get these two terrain features around the given grid in the 2.5D elevation map, it is necessary to search all the points inside the sphere centered at that grid. Flow chart of the searching algorithm is shown in Figure 5.

The algorithm searches the grids around the grid G(i,j) according to the radius $r_{normal}$, and the points belonging to such grids are added to the resulting point cloud $P_{ij}^{normal}$ step by step. Let $P_{ij}^{normal}$ be an ordered set composed of w points:

(12) $$P_{ij}^{normal}=\left\{p_{1}^{ij}\left(x_{1}^{ij},y_{1}^{ij},z_{1}^{ij}\right),p_{2}^{ij}\left(x_{2}^{ij},y_{2}^{ij},z_{2}^{ij}\right),\ldots ,p_{w}^{ij}\left(x_{w}^{ij},y_{w}^{ij},z_{w}^{ij}\right)\right\}.$$

Principal component analysis is used to compute the normal and curvature from the set $P_{ij}^{normal}$, a medial matrix A with the size w×3 is firstly constructed:

(13) $$A=\left[\begin{array}{ccc} x_{1}^{ij}-\bar{x^{ij}}\; & \;y_{1}^{ij}-\bar{y^{ij}}\; & \;z_{1}^{ij}-\bar{z^{ij}},\\ x_{2}^{ij}-\bar{x^{ij}}\; & \;y_{2}^{ij}-\bar{y^{ij}}\; & \;z_{2}^{ij}-\bar{z^{ij}},\\ \vdots \; & \;\vdots \; & \;\vdots \\ x_{w}^{ij}-\bar{x^{ij}}\; & \;y_{w}^{ij}-\bar{y^{ij}}\; & \;z_{w}^{ij}-\bar{z^{ij}} \end{array}\right].$$

In Equation (13), the average value of 3D coordinates is computed as the following equation:

(14) $$\left\{\begin{array}{c} \bar{x^{ij}}=\frac{1}{w}\times \left(x_{1}^{ij}+x_{2}^{ij}+\ldots +x_{w}^{ij}\right),\\ \bar{y^{ij}}=\frac{1}{w}\times \left(y_{1}^{ij}+y_{2}^{ij}+\ldots +x_{w}^{ij}\right),\\ \bar{x^{ij}}=\frac{1}{w}\times \left(x_{1}^{ij}+x_{2}^{ij}+\ldots +x_{w}^{ij}\right). \end{array}\right.$$

Now, the covariance matrix $C_{ij}^{normal}$ of the 3D point cloud $P_{ij}^{normal}$ can be computed from the medial matrix A:

(15) $$C_{ij}^{normal}=A^{T}\times A.$$

$C_{ij}^{normal}$ can be decomposed by eigenvalue:

(16) $$C_{ij}^{normal}\times \left[\alpha_{1},\alpha_{2},\alpha_{3}\right]=\left[\begin{array}{ccc} \lambda_{1}\;\; & \;\;\;\; & \;\;\\ \;\; & \;\;\lambda_{2}\;\; & \;\;\\ \;\; & \;\;\;\; & \;\;\lambda_{3} \end{array}\right]\times \left[\alpha_{1},\alpha_{2},\alpha_{3}\right].$$

$\lambda_{i}$ is the eigenvalue of the covariance matrix $C_{ij}^{normal}$, and $\alpha_{i}$ is the eigenvector with respect to the eigenvalue $\lambda_{i}$. Suppose that the eigenvalues satisfy the ordering constraint: $\lambda_{1}<\lambda_{2}<\lambda_{3}$, according to the theory of principal component analysis, $\alpha_{1}$ is the normal vector of the terrain at grid G(i,j), as shown in Equation (17):

(17) $$n^{ij}={\left[n_{x}^{ij},n_{y}^{ij},n_{z}^{ij}\right]}^{T}=\alpha_{1}.$$

The curvature is represented by the normalization of minimum eigenvalue shown as Equation (18):

(18) $$C_{urv}\left(i,j\right)=\frac{\lambda_{1}}{\sqrt{{\lambda_{1}}^{2}+{\lambda_{2}}^{2}+{\lambda_{3}}^{2}}}.$$

## 4. Designing the Cost Function of Selecting a Foothold

### 4.1. Rules for Selecting a Foothold

The best foothold selected should satisfy the following rules as much as possible: 

(1) Foot slip should be avoided or minimized.

(2) Walking speed should be maximized. For two candidate footholds, one lies at the front of the default foothold, and the other lies at the back; the priority of the front foothold should be larger than that of the back one.

(3) The distance between the candidate foothold and default foothold should be minimized, according to the law of quadruped bionic movement.

(4) The area of the supporting triangle should be maximized for increasing the stability of robot walking.

It is impossible to meet all requirements simultaneously. Even some rules are contradictory, it is therefore necessary to weigh such rules into consideration. It is not possible to weigh such rules by adjusting parameters manually, so expert learning strategy is employed to consider those rules comprehensively.

**Figure 6:**
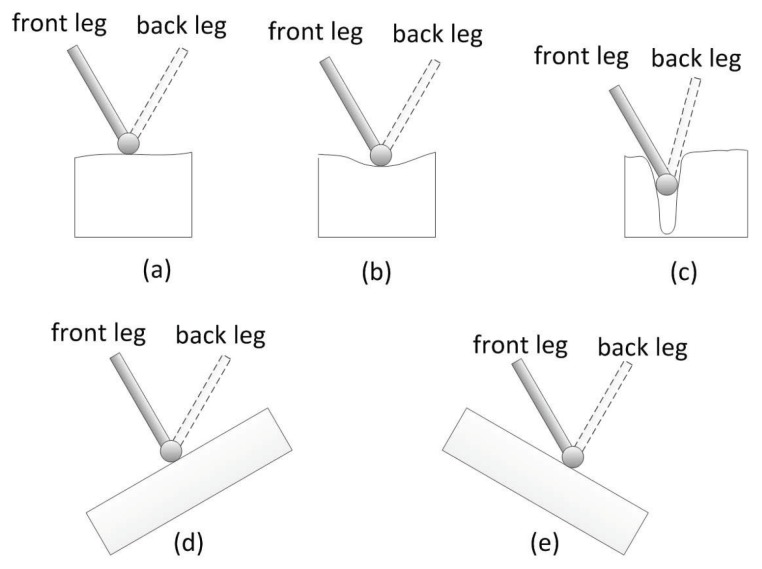
Several classical contact models between foot and terrain.(**a**) shows the case of flat terrain; (**b**) shows the case of terrain with little curvature; (**c**) shows the case of terrain with large curvature; (**d**,**e**) show the preferences of selecting foothold for front and back leg, respectively.

In order to avoid sliding of the foot from the ground as much as possible, some classical contact models between the foot and ground are listed, which are shown in Figure 6. Front and back legs are configured differently in the figure, in terms of the view used in Figure 1.

In the first three cases (a)–(c), the normal of the terrain is vertical to the horizontal plane, but there is some angle between the normal of the terrain and the horizontal plane in the cases (d) and (e), with uphill and downhill slope, respectively. For the first three cases, priority of model (b) is the highest, no matter which leg it is, because the curvature of the terrain in model (b) is larger than that of (a). Priority of model (c) is lower than that of (a) because the curvature of the terrain in model (c) is too large to lift the foot smoothly. In our experiment, the foothold with curvature larger than 0.16 must be excluded from the candidate set. For the last two models (d) and (e), selection characteristics of front legs are different that of back legs, which is determined by the robot structure; feet of the front legs are used to step down forwardly, while that of the back leg is used to step down backwardly. Thus, the terrain with an uphill slope is more suitable for the front foot, while the terrain with a downhill slope is more suitable for the back foot.

### 4.2. Cost Function Definition

Only the candidate footholds around the default foothold are comparable. Suppose there are *n* candidate footholds in the rectangle centered at the default foothold, as shown in Equation (19), in which $\xi_{i}$ is the feature vector of the *i*th candidate foothold:

(19) $$F_{c}=\left\{\xi_{1},\xi_{2},\ldots ,\xi_{i},\ldots ,\xi_{n}\right\}.$$

The cost of selecting one foothold whose feature vector belongs to the set $F_{c}$ will be computed using the cost function, and the foothold with the lowest cost would be selected. The feature vector of each foothold is 11-dimensional. In Equation (20), $\lambda_{j}$ is the *j*th element of the feature vector:

(20) $$\xi ={\left[\lambda_{1},\lambda_{2},\ldots ,\lambda_{j},\ldots ,\lambda_{11}\right]}^{T}.$$

The feature vector is composed of both the terrain features and positional relationship between the candidate foothold $p_{c}$ and the default foothold $p_{d}$.

The terrain features are listed below:

(1) $f_{1}^{t}$: The curvature of the terrain around the candidate foothold $p_{c}$ is used to judge whether the terrain is flat or not, which is considered for decreasing the foot slip.

(2) $f_{2}^{t}$: The slope of the terrain around the candidate foothold $p_{c}$ is computed as the angle between the normal of the terrain centered at $p_{c}$ and the horizontal surface.

(3) $f_{3}^{t}$: The feature for characterizing the terrain is concave or convex. Let $p_{1},p_{1},\ldots ,p_{8}$ have eight points with uniform distribution around the candidate foothold $p_{c}$, and $v_{i}$ is the directed vector from $p_{c}$ to $p_{i}$, where i∈[1,8]. The intersection angle between $v_{i}$ and the normal of the terrain centered at the point $p_{c}$ is computed, and then all eight of the angles are averaged as the feature value. The concave terrain is better than the convex one, as long as the foot can not be stuck.

(4) $f_{4}^{t}$: The feature for characterizing the terrain is uphill or downhill. The vertical plane P is constructed based on the walking direction of the robot D and the vertical upward axis Z. Suppose $n^{\prime }$ is the vector which is projected onto the plane P from the terrain normal n at the candidate foothold. The rotational angle from D to $n^{\prime }$ is computed as the feature value. It is very important to judge that the terrain is uphill or downhill because the front foot of the quadruped robot prefers uphill terrain, while the back foot prefers downhill terrain.

Both the normal and curvature are estimated based on the 3D points inside the sphere with a radius $r_{normal}$ as shown in Figure 4, so the terrain features will change each time the radius is adjusted. In order to get terrain information with different scales, two radiuses are employed, 2 cm and 5 cm, respectively. $\lambda_{1}-\lambda_{4}$ and $\lambda_{5}-\lambda_{8}$ in the feature vector ξ are terrain features $f_{t1}-f_{t4}$ computed with the radius 2 cm and 5 cm, respectively.

Position related features of the vector:

(1) $f_{1}^{p}$: The feature for representing the distance between the candidate foothold and the default foothold, which will make the quadruped robot select the foothold closer to the default foothold as much as possible.

(2) $f_{2}^{p}$: The feature for characterizing that the candidate foothold $p_{c}$ lies on the left side of the default foothold or the right side. Let us define the rectangular coordinate system $O_{d}$, its original point is $p_{d}$, which is the projection of the default foothold. The X-axis direction is the same as D, which is the walking direction of the robot, the Y-axis is vertical with X and lies at the left side of the axis X, the coordinate value of $p_{c}$ w.r.t Y is computed as the feature value.

(3) $f_{3}^{p}$: The feature for illustrating that the candidate foothold $p_{c}$ lies on front of the default foothold or back of it. The coordinate value of $p_{c}$ w.r.t X in system $O_{d}$ is computed as the feature value.

In fact, the positional relationship $f_{1}^{p}-f_{3}^{p}$ represents a polar coordinate in the coordinate system, in which the pole is the point $p_{d}$, and the polar axis is the moving direction of the robot. These three features are used for weighing the last three rules of selecting the foothold. $\lambda_{9}-\lambda_{11}$ in the feature vector ξ is the positional relationship $f_{1}^{p}-f_{3}^{p}$.

The weight vector W is defined in Equation (21), and the dimensions of the vector are the same as that of the feature vector:

(21) $$W={\left[\omega_{1},\omega_{2},\ldots ,\omega_{j},\ldots ,\omega_{11}\right]}^{T}.$$

Now, the cost function is defined as the weighted sum of feature values:

(22) $$C_{foot}\left(\xi \right)=W^{T}\xi =\sum_{k=1}^{11}\omega_{k}\lambda_{k}.$$

## 5. Learning the Weight Vector of the Cost Function

In order to get the cost function $C_{foot}\left(\xi \right)$, it is a key step to estimate the weight vector as Equation (21) w.r.t some quadruped robot and terrain environment. The weight vector is also called parameters of the cost function.

### 5.1. Training Data Collection

The learned cost function will be applied to navigate the real quadruped robot, so the training data set for learning the weight vector of the cost function must be collected in some type of terrain, on which the quadruped robot will walk.

**Figure 7:**
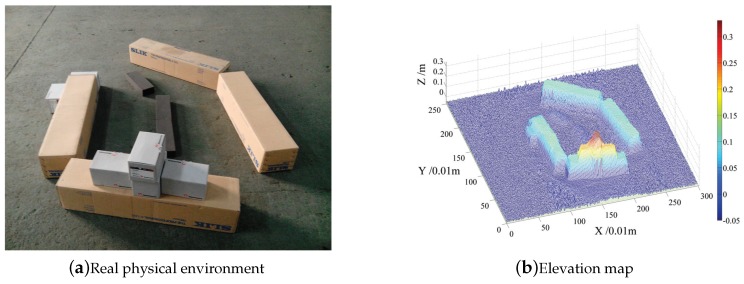
Terrain model for collecting training data set. (**a**) is the real terrain environment and (**b**) is the corresponding 2.5D elevation map constructed from 3D point cloud; several frames are captured around the terrain model, and they are registered using a modern SLAM algorithm [4,5,6].

Given the real terrain shown in Figure 7a, its 2.5D elevation map as Figure 7b is constructed from the point cloud generated by applying TOF camera, the colorbar in Figure 7b indicates the elevation value, and then the training data set is collected based on the elevation map. A simulated quadruped robot with the same kinematic model as the real robot is placed randomly on the map. Four candidate footholds are generated randomly around the default foothold. The expert sorts four such candidate footholds, allowing feature vectors of the four footholds shown in Equation (23). Suppose that the first foothold is the best one, the fourth foothold is the worst one, and the second foothold is better than the third one. The smaller the value of cost function, the better the foothold is. Sorted data like Equation (24) will be collected as the training data, where W is the weight vector to be learned and $\xi_{i}$ is the feature vector of the *i*th foothold.

(23) $$F_{c}^{4}=\left\{\xi_{1},\xi_{2},\xi_{3},\xi_{4}\right\},$$

(24) $$W^{T}\xi_{1}\leq W^{T}\xi_{2}\leq W^{T}\xi_{3}\leq W^{T}\xi_{4}.$$

When the expert gives ranks of these footholds, several rules should be obeyed:

(1) Priority of the candidate foothold with a shadow pit is higher;

(2) Priority of the candidate foothold with a deep slope is lower;

(3) Priority of the candidate foothold will lead to the collision between the leg and terrain being lower;

(4) Priority of the candidate foothold at the edge of the terrain is lower;

(5) The candidate foothold with the possibility of sliding is preferred rather than that with the danger of the leg sticking;

(6) The five rules above an elaborate influence of the terrain to foothold selection, for the two candidate footholds with similar terrain features, the one with a closer distance from the default foothold has higher priority;

(7) The candidate foothold lying at the front of the default foothold has higher priority; suppose that the six principles above are similar for two candidate footholds;

(8) The candidate foothold maximizing the next supporting triangle has higher priority; suppose that the seven principles above are similar for two candidate footholds.

**Figure 8:**
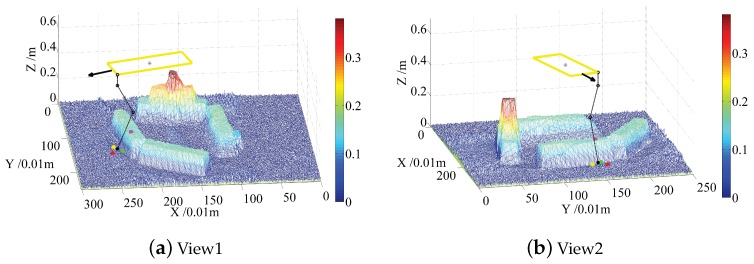
Candidate footholds randomly sampled around the ***left front*** default foothold for experts, and experts will sort these footholds by looking from several clear views. (**a**) the first view from which the expert can judge that the candidate foothold lies at the front of the default foothold or back; (**b**) the second view from which the expert can judge that the candidate foothold lies to the left of the default foothold or right.

**Figure 9:**
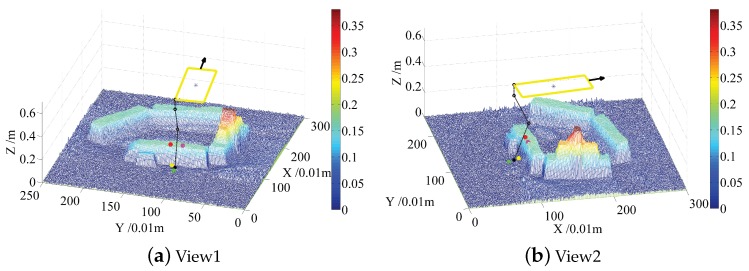
Candidate footholds randomly sampled around the ***left back*** default foothold for expert, and expert will sort these footholds by looking from several clear views. (**a**) the first view from which the expert can judge that the candidate foothold lies to the left of the default foothold or right; (**b**) the second view from which the expert can judge that the candidate foothold lies at the front of the default foothold or back.

In order to explain the rules above vividly, Figure 8 and Figure 9 show the views of the default footholds and candidate footholds for left front, and left back, respectively. The two cases shown in Figure 8 and Figure 9 are typical because collision is prone to happen when the front foot moves down a step, and that the back foot moving up a step is also prone to colliding. For each case, several views can be shifted by experts to sort candidate footholds accurately; here, only two views are shown for saving paper space. The blue star lies at the center of the robot body, the black sphere on the map stands for the default foothold, the black line with arrows indicates the moving direction. Four sampled candidate footholds are represented by spheres with green, red, yellow and purple colors, respectively.

For the case in Figure 8, the priority order of these four candidate footholds is represented using the color of foothold: red⪰yellow⪰purple⪰green. The red foothold has the highest priority because it lies in the left front side of the default foothold, which will make the triangle area larger and the walking faster. The worst foothold is the green one, due to collisions between legs and terrain. The yellow foothold is better than the purple one because the yellow foothold is closer to the default foothold, and the purple foothold lies at the back of the default foothold, which will decrease the walking speed.

For the case in Figure 9, priority order of these four candidate footholds is represented using the color of foothold: red⪰purple⪰green⪰yellow. Priorities of the red and purple footholds are higher than that of green and yellow footholds because the former two footholds lie at the front of the default foothold. The yellow foothold is the worst one due to collisions between legs and terrain. The red footholds lie on the left side of the default foothold, which will increase triangle area, but the purple one lies on the right side, so the red foothold is better than the purple one.

### 5.2. Weight Vector Computation

The front foot and the back foot do not prefer the candidate foothold with the same feature vector, according to Figure 6. The left foot and the right foot also have different preferences for considering stability of the robot. Thus, the cost functions w.r.t. four feet are different, and the weight vector needs to be optimized individually.

Once four candidate footholds are generated on the elevation map, the order of the footholds is given by the expert, and then the order, the terrain feature and status of the robot are recorded simultaneously. n(=1000) groups of data are recorded for one foot, and that of other three feet are recorded using the same approach. The training data sets are represented by $D_{train}^{LF},D_{train}^{RF},D_{train}^{LB},D_{train}^{RB}$ for four feet, respectively. The weight vector is learned based on the training data set using a support vector machine for each foot, and four vectors are represented by $W_{LF},W_{RF},W_{LB},W_{RB}$, respectively, which are shown in Table 4.

In order to validate the reasonableness of the learned weight vector, it is used to reorder each group of candidate footholds in the training data set. There are $C_{4}^{2}$ ordered pairs of candidate footholds in each group, so $1000C_{4}^{2}$ ordered constraints can be provided by the expert for each foot. If *m* ordered foothold pairs are computed from the cost function of candidate footholds using Equation (22), then the accuracy of the weight vector is $m/\left(1000C_{4}^{2}\right)$, shown in Table 5 for each foot.

## 6. Experimental Results and Discussion

In this section, semi physical and physical experiments are both carried out to validate the effectiveness of the proposed cost function. The cost function is applied on the offline elevation map, which is used to learn the parameters of function in the semi physical experiment. Furthermore, in the physical experiment, the quadruped robot selects a suitable and safe foothold using the cost function on the elevation map constructed in real time.

### 6.1. Semi Physical Experiment

**Figure 10:**
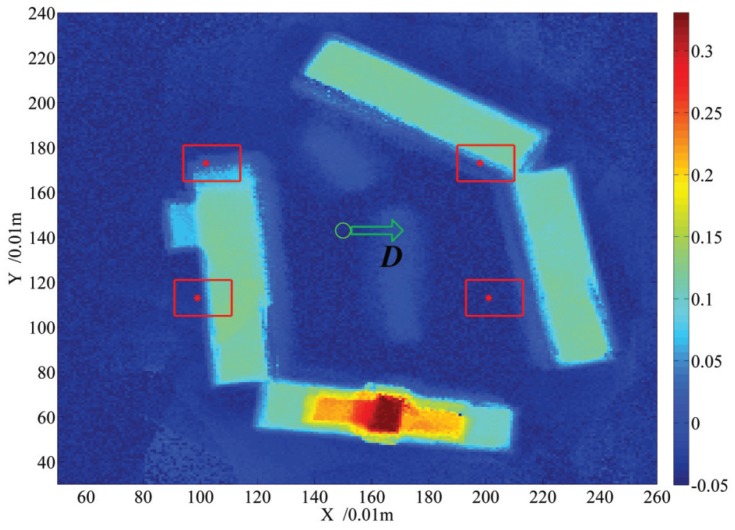
Four default footholds computed from the robot body center and moving direction, a region of candidate footholds around a default foothold is also marked using a red rectangle for each foot, respectively.

A body center point is selected on the 2.5D elevation map shown in Figure 7b, and then four default footholds are computed using the Equation (4), given that the quadruped robot walks along the the forward direction of the X axis. For viewing clearly, the elevation map and the footholds are projected into 2D space, which is shown in Figure 10. Elevation value is represented by the color, the green circle is the robot center, the red circle dots are default footholds, the green arrow stands for the walking direction and candidate footholds lie inside the red rectangle. Coordinates of the default footholds are listed in Table 6 in two units: *m* and grid. The candidate region on the front of the default foothold is larger than that on the back, which ensures the robot walking forward, to some extent.

**Figure 11:**
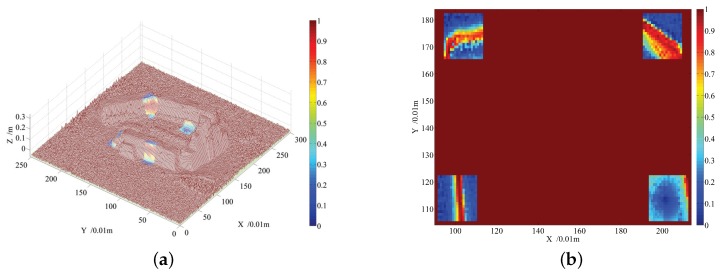
The cost map for candidate footholds in the real world. (**a**) Cost of candidate footholds represented in a 2.5D elevation map. (**b**) Cost of candidate footholds represented in the projected 2D map.

For each grid inside the red rectangle region, cost is computed using the cost function shown in Equation (22), given the learned weight vector in Table 4, the cost value is represented by the color, which is shown in Figure 11. For the candidate footholds outside all the rectangles, their costs are set to the maximized value 1 because only the footholds inside the same rectangle are comparable for the cost value. According to the experimental results shown in Figure 11, the cost of the candidate foothold lying at the edge of the terrain is larger, which can be clearly viewed by referencing the 2D map in Figure 11b. For the flat region inside the lower right rectangle in Figure 11b, which is the candidate region for the front right foot of the quadruped robot, the costs of the grids around the default foothold are lower than that far from the default foothold. For the two grids with the same distance from the default foothold, the grid lying in the lower right has a smaller cost because it can make the supporting triangle larger, which is carried out by obeying the rules for selecting a foothold presented in Section 4. The semi experimental result demonstrates the effectiveness of the cost function for indicating the complexity of the terrain. In order to improve the fault tolerance of selecting a proper foothold for the quadruped robot, the average cost of the grids inside the circle centered at some grid is computed, and the grid with the lowest average cost is selected as the foothold in the experiment.

### 6.2. Experiments for Walking across a Step

Cost function is used to select footholds in real time for the quadruped robot (shown in Figure 1) walking across a wooden step. A target of the experiment is that the robot can walk across the step autonomously, and the width of the step is 30 cm, the height is 10 cm. The robot is controlled to walk based on the learned model of foothold selection, maintaining the robot body level up. The TOF camera is the only sensor to construct the map and position it on the map.

**Figure 12:**
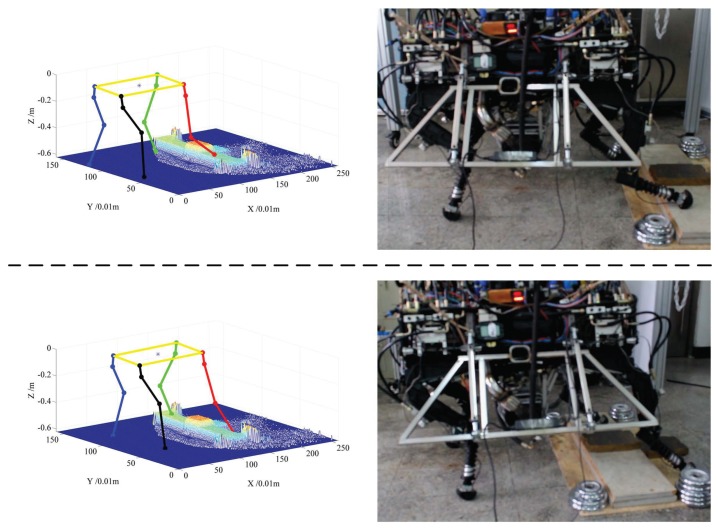
Screenshots of walking across the step, and corresponding poses of the robot on the elevation map, the upper panel shows a case of lifting and placing **the right front foot** on the step, and the lower panel shows cases of putting the foot onto the ground.

**Figure 13:**
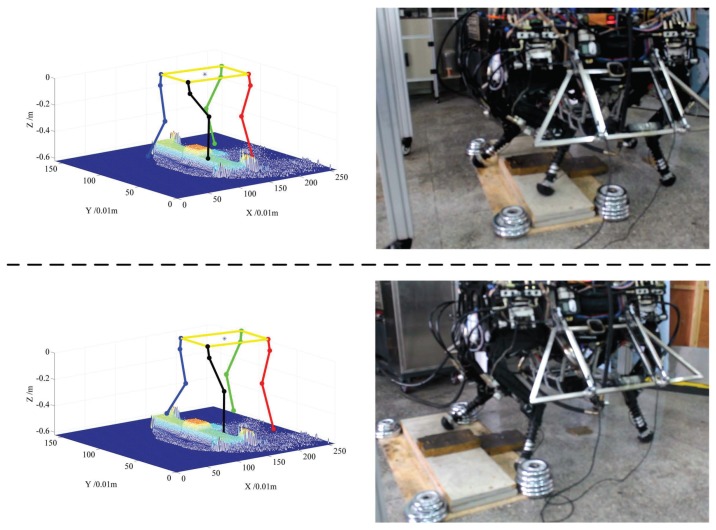
Screenshots of walking across the step, and corresponding poses of the robot on the elevation map, the upper panel shows a case of lifting and placing **the right rear foot** on the step, and the lower panel shows cases of putting the foot onto the ground.

Figure 12 and Figure 13 show the cases of walking across the step for right front foot and right back foot, respectively. In each figure, the right panel is the video screenshot of walking up the step and down the step, and the left panel shows the real elevation map and the skeleton of the robot at that time. From two figures, we can see that the robot can select a suitable foothold when crossing the step, which demonstrates the effectiveness of the cost function learned from a real terrain model. Only key screenshots are shown for saving paper space, but the robot is really controlled autonomously to cross the step from start point to end point.

**Figure 14:**
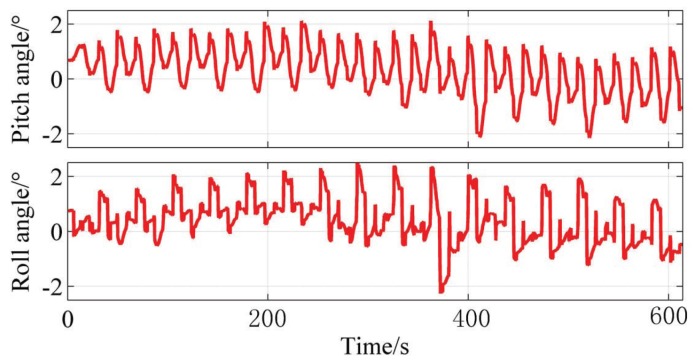
The intersection angle between robot base and horizontal plane.

In the experiment, the robot is set to maintain the robot body level, and Figure 14 shows the pitch and roll angle of the robot. The angle belongs to the range $\left[-2^{\circ },2^{\circ }\right]$, so the robot body is kept level more or less, which indicates the effectiveness of the controlling algorithm based on the TOF camera.

**Figure 15:**
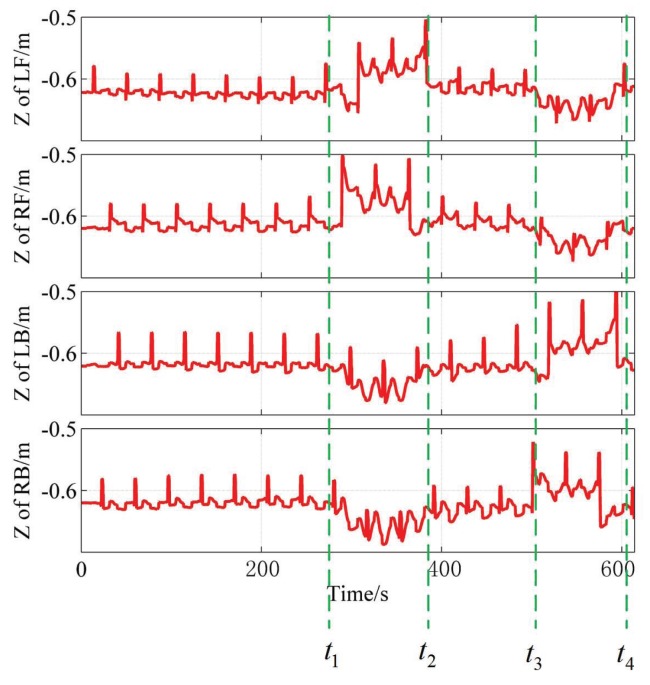
Z-coordinate of the foot in the coordinate system in the center of the robot body.

When the robot is walking on the ground, body height is set to 0.62 m; suppose the coordinate system of the robot body is located at zero w.r.t Z-axis, and then Z-coordinate values of four feet are rendered along the walking process which are shown in Figure 15. The whole time axis is partitioned manually using four green dotted lines. All four feet step on the ground for the time $\left[0,t_{1}\right)$; two front feet are crossing the step, while the two back feet step on the ground for the time $\left[t_{1},t_{2}\right)$; all four feet step on the ground for the time $\left[t_{2},t_{3}\right)$, although the step lies beneath the robot body in this time; the two back feet are crossing the step, while the two front feet step on the ground for the time $\left[t_{3},t_{4}\right]$.

**Figure 16:**
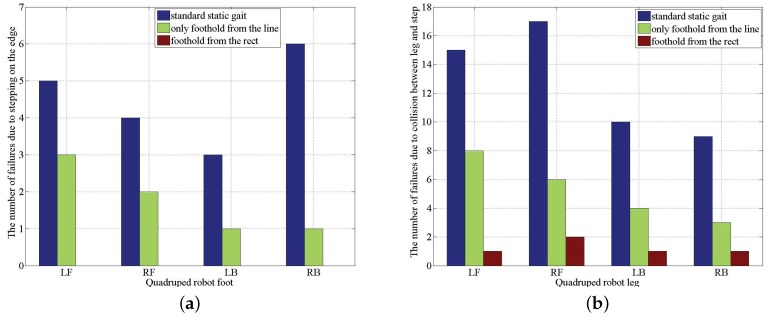
Comparison of the performance w.r.t. walking failure between the algorithm using standard static gait and that using a learned foothold selection. (**a**) The case of stepping on the edge of the wooden step. (**b**) The case of collision between the leg and the step.

In order to validate the effectiveness of the proposed algorithm for selecting a foothold, the previous experiment of a quadruped robot walking across the wooden step is carried out repeatedly, and three groups of the experiments are conducted 100 times, respectively. The wooden step is randomly placed in front of the robot in each experiment. In the first group of experiments, the standard static gait is applied, and the TOF camera is also used to map the wooden step and terrain in front of the robot and locate the robot on the map, which can keep walking with a horizontal robot body. In the second and third group of experiments, the learned foothold selection model is applied, and the candidate footholds come from the whole rect w.r.t. some default foothold in the third group, but, in the second group of experiments, the candidate footholds come only from the line crossing the default foothold and having the same direction with the walking direction D. The last two groups demonstrate that the candidate foothold on the lateral side of the default foothold will be better. The walking failure rate of the first group of experiment is 69%, the second group is 28%, and the last group is merely 5%, which shows the great advantage of the proposed model for selecting footholds. In general, the main results for failing to walk across the step include: (1) stepping on the edge of the step, which will lead to the foot sliding; and (2) collisions between the leg and step. The detailed comparison results are represented in Figure 16.

From Figure 16, it is easy to see that the probability of stepping on the edge is much lower than that of collisions between the leg and step, which is obvious because there are many more candidate footholds that can lead to collision. There are five failures walking across the wooden step. Even if the proposed model for selecting a better foothold is integrated into the controlling system, which is caused by the error of the model itself, the error of the TOF sensing and the foot sliding is due to the instability of the robot body, etc. The error of the model can be further reduced by increasing the training data.

## 7. Conclusions

This article presents a cost function for foothold selection in a quadruped robot that is equipped with a TOF camera. Through experimental results, we can see that the cost function is very effective for the robot to select a suitable and safe foothold. The cost function is defined as the weighted sum of the elements in the feature vector including both terrain features and other features related to the default foothold. The reasons for the excellent performance of the cost function include:

(1) The default foothold is introduced to the cost function, only candidate footholds around the default foothold are selected, and the candidate that makes larger supporting region area is given higher priority, which follows the walking regularity of the four-footed animal.

(2) The weight vector of the cost function is learned under the guidance of the expert, and the training data are collected on the 2.5D elevation map of a real terrain with several steps, so the quadruped robot always selects a proper foothold for the whole process crossing the terrain with steps.

The cost function can be extended to the quadruped robot walking a more rough terrain, as long as training data set with respect to such type of terrain is collected, on which our future research will be the focus.

## Figures and Tables

**Table 1:** Constant parameters of the model.

a(m)	b(m)	c(m)	d(m)	e(m)
0.5007	0.175	0.0852	0.31	0.3557

**Table 2:** Ranges for the joint angle of the right back leg.

$\alpha^{\mathrm{RB}}\left({}^{\circ }\right)$	$\beta^{\mathrm{RB}}\left({}^{\circ }\right)$	$\gamma^{\mathrm{RB}}\left({}^{\circ }\right)$
[−42.6426,31.0461]	[14.1359,120.3034]	[81.5307,180.7252]

**Table 3:** DH parameters for the right back leg.

*i*	$a_{i}\left(m\right)$	$d_{i}\left(m\right)$	$\theta_{i}\left({}^{\circ }\right)$	$\alpha_{i}\left({}^{\circ }\right)$
1	−b	0	0	90
2	−c	−a	$\theta_{2}=90+\alpha^{RB}$	90,
3	−d	0	$\theta_{3}=-90+\beta^{RB}$	0,
4	−e	0	$\theta_{4}=180-\gamma^{RB}$	0.

**Table 4:** Weight vector table.

Index	$W_{\mathrm{LF}}$	$W_{\mathrm{RF}}$	$W_{\mathrm{LB}}$	$W_{\mathrm{RB}}$
1	$3.78\times 10^{-5}$	$1.77\times 10^{-5}$	$3.86\times 10^{-6}$	$7.87\times 10^{-6}$
2	$3.46\times 10^{-4}$	$2.90\times 10^{-4}$	$4.60\times 10^{-4}$	$4.67\times 10^{-4}$
3	$-1.91\times 10^{-4}$	$-1.95\times 10^{-4}$	$4.83\times 10^{-5}$	$9.17\times 10^{-6}$
4	$1.46\times 10^{-4}$	$-3.89\times 10^{-5}$	$-1.65\times 10^{-4}$	$1.37\times 10^{-4}$
5	$1.88\times 10^{-5}$	$2.99\times 10^{-5}$	$2.63\times 10^{-5}$	$2.58\times 10^{-5}$
6	$3.39\times 10^{-4}$	$3.88\times 10^{-4}$	$3.68\times 10^{-4}$	$4.65\times 10^{-4}$
7	$-0.98\times 10^{-4}$	$-2.17\times 10^{-4}$	$-2.37\times 10^{-5}$	$-8.65\times 10^{-6}$
8	$9.56\times 10^{-5}$	$3.25\times 10^{-5}$	$-1.29\times 10^{-4}$	$-1.48\times 10^{-4}$
9	$1.68\times 10^{-5}$	$3.76\times 10^{-6}$	$1.58\times 10^{-5}$	$-8.64\times 10^{-6}$
10	$1.67\times 10^{-6}$	$-1.13\times 10^{-5}$	$-6.89\times 10^{-6}$	$-1.66\times 10^{-5}$
11	$1.70\times 10^{-6}$	$4.67\times 10^{-6}$	$5.77\times 10^{-6}$	$1.33\times 10^{-5}$

**Table 5:** The accuracy of weight vector on training data.

$W_{\mathrm{LF}}$	$W_{\mathrm{RF}}$	$W_{\mathrm{LB}}$	$W_{\mathrm{RB}}$
92.98541%	93.8%	91.145633%	94.24623%

**Table 6:** Coordinates and grids of the default foothold in the real world.

Format	$F_{\mathrm{lf}}$	$F_{\mathrm{rf}}$	$F_{\mathrm{lb}}$	$F_{\mathrm{rb}}$
Grids	$\left(198,173\right)$	$\left(201,113\right)$	$\left(102,173\right)$	$\left(99,113\right)$
Coordinates	(1.98 m, 1.73 m)	(2.01 m, 1.13 m)	(1.02 m, 1.73 m)	(0.99 m, 1.13 m)
